# Properties and Characterization of Cryogels: Structural, Mechanical, and Functional Insights

**DOI:** 10.1021/acsomega.5c02863

**Published:** 2025-08-11

**Authors:** Era Jain, Kaixiang Zhang, Ruchi Mishra Tiwari

**Affiliations:** † Department of Biomedical and Chemical engineering, 2029 Syracuse University, Syracuse, New York 13244, United States; ‡ Bioinspired Syracuse: Institute for Material and Living System, 2029 Syracuse University, Syracuse, New York 13244, United States; § Symbiosis Centre for Stem Cell Research, 29630 Symbiosis International (Deemed University), Pune 412115, India; ∥ Symbiosis School of Biological Sciences (SSBS), Symbiosis International (Deemed University), Pune 412115, India

## Abstract

Cryogels are a distinct class of macroporous polymeric materials formed through cryopolymerization, where precursor monomers and polymers undergo polymerization and cross-linking under freezing conditions. Unlike conventional hydrogels, which exhibit nanoscale porosity and are synthesized at ambient temperatures, cryogels feature interconnected micrometer-sized pores that confer unique mechanical, structural, and functional properties. Their high porosity, rapid hydration, and efficient mass transport make them highly desirable for tissue engineering, biosensing, drug delivery, and environmental remediation applications. However, a critical challenge remains a comprehensive understanding of the intricate relationships among synthesis parameters, microstructure, and functional performance. This review provides a systematic discussion of cryogel properties, with a focus on their mechanical resilience, biocompatibility, and shape recovery behavior. We examine recent advancements in characterization techniques, including in situ imaging, advanced rheological assessments, and machine learning-assisted porosity evaluation, which have significantly improved our ability to assess cryogel performance. Additionally, we review the biophysical characterization of cryogels composed of different polymer systems, elucidating structure–property correlations in pore architecture and cellular interactions. Expanding beyond traditional biomedical applications, we briefly describe the emerging potential of cryogels in biosensors, soft robotics, and environmental sustainability, emphasizing the importance of an integrated approach that links the structure to functional outcomes. By providing a detailed discussion of established and cutting-edge characterization methodologies, this perspective is a valuable resource for researchers striving to develop next-generation cryogels with precisely tailored properties for specialized applications.

## Graphical abstract

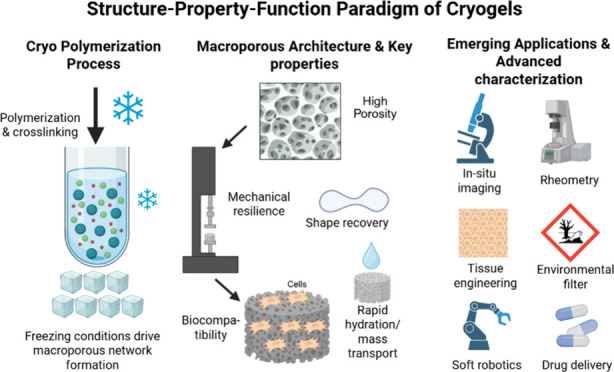

## 1 Introduction

Cryogels are a unique group of macroporous polymeric scaffolds developed by polymerization and cross-linking of precursors under freezing conditions.^1,2^ The process of cryogel synthesis involves choosing proper monomers, cross-linkers, and initiators, followed by their mixing to form a homogeneous phase. The solution is incubated at subzero temperatures, wherein the partial freezing of the aqueous solvent into ice occurs, forming a heterogeneous phase of ice-crystals and the monomers, cross-linkers, and initiators restricted to the unfrozen liquid microphase. Here, the ice crystals create the template for the porous structure.^3,4^ Simultaneously, the continuous unfrozen liquid phase represents the site of a developing polymer network during the proceedings of the polymerization reaction.^5–7^ After incubation, the ice crystals are melted away, creating a highly interconnected, sponge-like three-dimensional macroporous network.^8,9^ A diagrammatic rendering of cryogel synthesis representing the three stages of cryogel formation as discussed above is shown in Figure 1.

**Figure 1:**
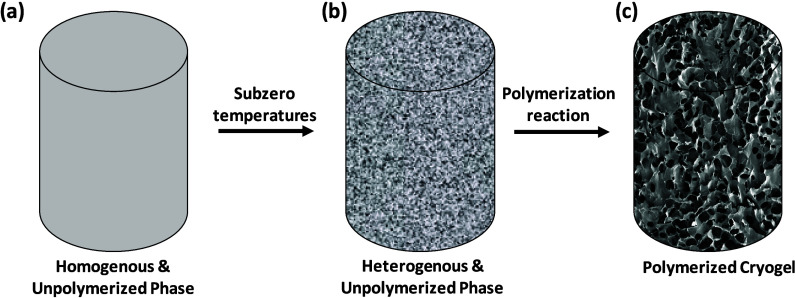
Diagrammatic rendering of the process of cryogel formation.

Unlike conventional hydrogels, which generally exhibit nanoscale porosity and limited mass transport capabilities and are synthesized at ambient conditions, cryogels features a combination of mechanical robustness, rapid hydration, and efficient molecular and cellular exchange.^8,10,11^ The unique combinations of porosity, injectability and ease of handling have led to widespread use of cryogels across multiple fields, particularly tissue engineering, drug delivery and regenerative medicine^12–29^ Smart cryogels having the capability to respond to changes in pH, temperature, or biochemical signals are being developed for programmed drug release, one-step cell purification, biosensing and environment remediation.^3,15^ Additionally, progress in cryogel fabrication techniques, including additive manufacturing and microfluidic-assisted cryogelation, has enabled more precise control over pore architecture and mechanical properties, broadening their applicability in personalized medicine and advanced biomaterials design.^30^

Despite these advancements, a comprehensive understanding of the intricate relationships among synthesis parameters, microstructural features, and functional outcomes remains limited. The mechanical performance, swelling behavior, pore architecture, and interaction with biological systems are all strongly influenced by variables such as monomer concentration, cryopolymerization temperature, freezing rate, and nature of the polymer backbone. Without a systematic framework linking these synthesis variables to resultant material properties, the rational design of cryogels for application-specific use remains a challenge. This review aims to offer a systematic discussion on the fundamental properties of cryogels, their underlying structure–function relationships, and the latest characterization techniques used to evaluate their structure and performance. The novelty of this review is highlighted in the holistic approach of connecting the chemical and physical aspects with the applicability of the cryogels. We also discuss recent advancements that have expanded the scope of cryogels beyond traditional biomedical applications, investigating their potential in evolving fields such as soft robotics and environmental sustainability. Additionally, we discuss the latest methodological advancements in cryogel characterization, particularly the integration of in situ imaging, advanced rheological analysis, and machine learning-assisted porosity evaluation. By providing a comprehensive discussion of both established and cutting-edge techniques, this review provides a critical resource for researchers aiming to design next-generation cryogels with tailored properties for specific applications.

## 2 Cryogel: Properties and Characterization

The process of cryogelation (polymerization under subzero temperatures) imparts unique properties to the cryogels which are not possible to achieve in conventional hydrogels synthesized at room temperature. The synthesis of cryogels under frozen conditions allows the creation of thick pore walls due to cryoconcentration of the precursors in the nonfrozen aqueous microphase. Whereas ice-crystals lead to pore formation upon postpolymerization thawing, leading to the formation of an open macroporous network that is a distinctive property of cryogels. The open network in cryogels allows for efficient mass transport of solutes of all sizes, including cell slurries.^7,31–33^ Cryogels are often associated with high elastic strength and extraordinary ability to hold water which makes them highly biocompatible.^34^ In this review, we further discuss in-depth the properties and methods that can be used for the extensive quantification and characterization of the cryogels.

### 2.1 Pore Structure, Porosity, and Interconnectivity

The main characteristic feature of cryogels is the interconnected web of pores with sponge-like morphology. Open interconnected pore structure in cryogels is also evident from their transport properties, as discussed in Section 2.1.2. The interconnected pore network of cryogels is an advantageous feature with respect to its use in different fields of tissue engineering,^15^ bioseparation,^35^ and cell immobilization matrix. The pore size in a typical cryogel may vary from 1 μm to −250 μm, depending upon the type of polymeric system and conditions used for the synthesis of the gel. Usually, oval-shaped pore formation is seen in cryogel made in aqueous solvent. However, pore shape and size are determined by the type of polymeric system and the crystal shape/size of the solvent used for synthesis. The effects of polymer precursor and solvent on gel synthesis and pore shape have been discussed in detail in some earlier reviews as cited here.^36^

#### 2.1.1 Techniques Used for Determination of Pore Structure, Porosity, and Interconnectivity

Due to the critical role of cryogel porosity in several applications, pore structure, density, and interconnectivity are studied using a combination of techniques. These include scanning electron microscopy (SEM),^37^ mercury porosimetery,^38^ and nitrogen adsorption isotherm.^39^ The SEM and mercury porosimetery measures pores in the nano- to micron-size range, while the nitrogen adsorption technique detects pores in the range 3–200 nm.^40^ Cryogels being soft, spongy, and highly hydrated in nature present a considerable challenge in the assessment of pore architecture by such techniques. A common limitation of all of these techniques is that the cryogels must be dried before these can be used for measurement. The drying results in loss of water, which is a major structural component of cryogel pore architecture. Moreover, mercury porosimetery involves the use of high pressure, which can lead to gel compression and thus inaccurate measurement of pore volume and sizes.^41^

To investigate cryogels in hydrated states, researchers have used techniques including cryo-SEM and environmental scanning electron microscopy (ESEM).^42^ Furthermore, ESEM allows visualization of the pore morphology as the cryogel dehydrates^43^ (Figure 2A). Moreover, both methods require minimal sample preparation. The microscopic images of cryogels taken via various imaging techniques are shown in Figure 2A–D.

**Figure 2:**
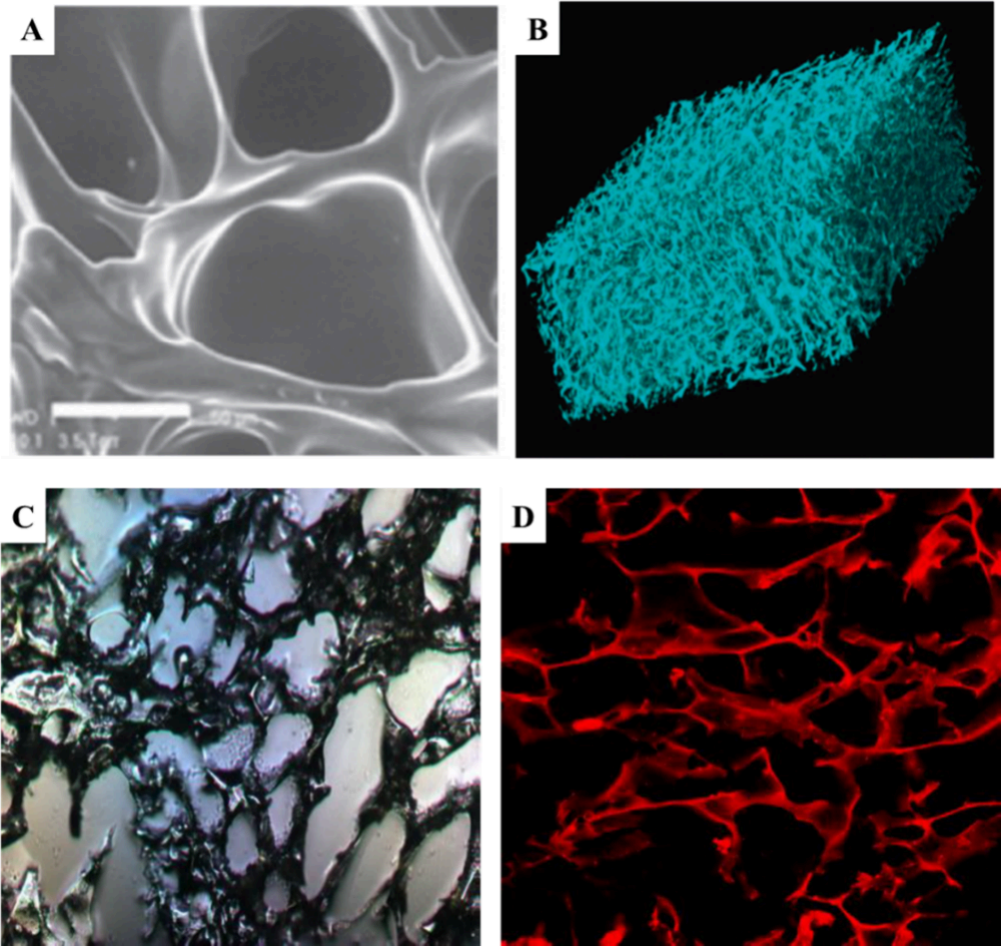
Cryogel pore structure imaged using various techniques. (A) Images of hydrated cryogel by ESEM adapted or reprinted in part with permission from cited ref^43^. Copyright [2005/Royal Society of Chemistry]. (B) 3D reconstruction of the μCT images of poly­(HEMA)-cryogel reproduced with permission from cited ref^46^. Copyright [2005/Royal Society of Chemistry]. (C) Optical image of 5% w/v poly­(*N*-isopropylacrylamide) (PNiPAAm)-chitosan cryogel; (D) 2D confocal image of 5% w/v polyacrylamide (PAAm) cryogel stained by eosin.

Additional in situ imaging techniques that allow measurement of the pore architecture in the wet condition are comprised of confocal laser scanning microscopy (CLSM), multiphoton microscopy, and X-ray microcomputed tomography (μ-CT). CLSM and multiphoton microscopy allow high-resolution imaging of fluorescently labeled cryogels (Figure 2D). However, CLSM microscopy is limited by the penetration depth; thus, only 200 μm thick sections can be studied.^44^ Two-photon fluorescence microscopy can be used to measure the pore size and pore-wall width in successive planes of fixed cryogel samples. These studies also demonstrated that cryoconcentration of the polymer during the freezing phase leads to the formation of dense pore walls.^45^ While, μCT allows for nondestructive 3D visualization of pore networks. A prestained sample can be scanned to generate a 3 D reconstruction and analyzed using ImageJ. μ-CT allows for scanning of relatively large cryogel samples, and 3D reconstructed images so obtained show a clear interconnected web of pores throughout the sample. An advantage of μ-CT over other techniques is that the interconnectivity of the pores can be quantified. It is expressed in terms of the number of independent entities that can be identified within the cryogel. Thus, each number denotes an independent pore not interconnected to other pores. Thus, a lower number denotes a high interconnectivity. During μ-CT analysis of poly­(2-hydroxyethyl methacrylate) (HEMA)-cryogels, a single object was shown, demonstrating one huge pore with interconnected channels (Figure 2B).^46^

Only limited techniques can be utilized for the assessment of soft, porous materials in their wet state. These materials are more likely to have very different properties in their hydrated state.^36^ Techniques, such as ESEM or NMR, can allow for measurement of pore size and porosity in their biological environment. While ESEM can visualize cryogel samples in the wet state (Figure 2A), it may not offer an accurate view of the inner pore structure. Indirect methods like solvent displacement, NMR, may not allow direct visualization of pores but can provide quantitative information related to cryogel porosity and pore architecture.

The solvent displacement method also gives an approximate measure of the pore volume of cryogels. Solvent displacement involves replacing the water in the cryogels with a nonsolvent like cyclohexane which displaces nonpolymer bound solvent. The volume of solvent used in the process gives an estimate of the porosity of the cryogels.^47^

Quantitative methods, including the thermogravimetric method, differential scanning calorimetry, ^1^H NMR cryoporometry, and thermally stimulated depolarization, are used to indirectly quantify the pore size and pore volume based on the state of water in cryogels. The water in cryogels may be present in three different states: nonbound water in the macropores, weakly bound water in the nanopores, and tightly bound water to the polymer surface (measures surface area). These different states of water give different signals or evaporate at different temperatures when analyzed by the above-mentioned techniques, giving an estimate of the amount of each type of water. For example, in NMR cryoporometry, the cryogel is dipped in a suitable solvent and is frozen. The sample is then slowly warmed, causing the solvent to melt. The quantity of released liquid is measured using NMR. The water in small pores or water bound tightly to the polymer melts at a lower temperature than nonbound water. The pore size and the depression of the melting point of water are inversely correlated.^47,48^ For a more advanced discussion of the methods to characterize pores and pore size distribution in the cryogels, please see the excellent review cited here.^48^

#### 2.1.2 Transport Properties of Cryogels

The large pore size and pore interconnectivity allow practically unhindered convective and diffusion transport of any solvent and solute particle of varying sizes through the cryogels.^49^ Flow rate or resistance to flow of the liquid through cryogels also gives an indirect estimate of the interconnected porous structure of the cryogels. The flow rate of solutions in the cryogel scaffolds is controlled by varying the precursor concentration and the parameters of the cryogenic treatment.^34^ In the case of polyacrylamide (PAAm) cryogels, a study shows that an increase in the total monomer concentration decreases the hydraulic permeability and that the effect of the cross-linker is highly distinct at low monomer concentrations (Figure 3A,B). Further low cross-linker to monomer concentration ratio leads to low hydraulic permeability owing to the collapse of pores under high shear stress (Figure 3B).^50^

**Figure 3:**
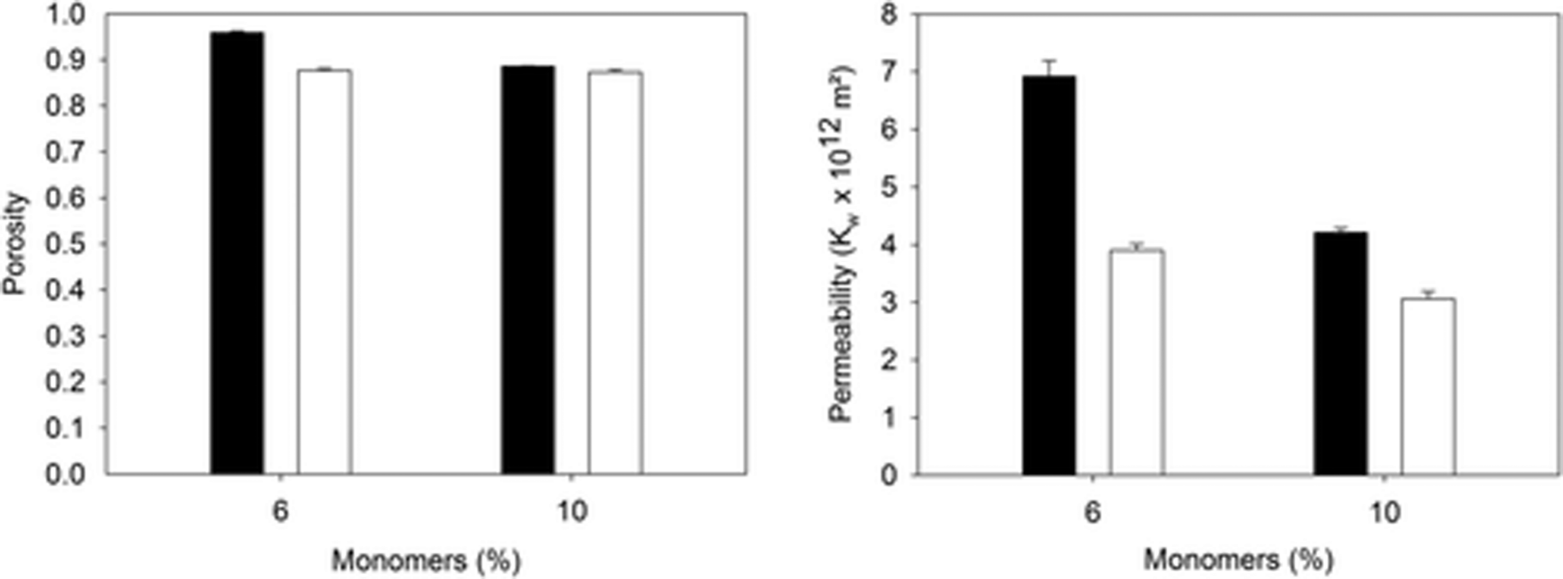
Porosity and permeability of cryogels are dependent upon the starting monomer concentration. The figure shows PAAm cryogels made using the indicated monomer concentrations with different monomer to cross-linker ratios of acrylamide/methylene bis-acrylamide (AAm/MBAAm ratios) (■4:1; □ 5:1)). Adapted or reprinted in part with permission from cited ref^50^. Copyright [2019/John Wiley and Sons].

Depending upon the synthesis parameters, the flow rate of aqueous solvent buffers through the cryogel matrix can vary between 0.5 and 10 mL/min.^49^ The unhindered flow-through of unpurified cell slurries through cryogels has led to special interest in their applications as chromatographic matrices in bioseparation.^4,49,51^ Unlike expanded bed chromatography, which requires specialized equipment, cryogels can directly process cell slurries without preclarification. Although fixed-bed chromatography is highly efficient, it typically requires preclarification of the cell broths before application onto the stationary bed. Due to the ability to process crude cell slurries, the use of cryogel columns has further expanded to classical fixed bed chromatography.^52^ These unique characteristics, combined with their easy-to-use format, make cryogels unique monolithic chromatography columns for protein purification. They can efficiently process particulate-containing cell suspensions and nonclarified mammalian cell suspensions without clogging or damaging the column itself.^53,54^

Bacterial cellulose containing poly­(HEMA)-poly­(vinyl alcohol) (pHEMA-PVA-BC) cryogels were prepared using a hybrid machine learning model. Of the four different models tested, the gradient boosted regression trees (GBRT) exhibited the best predictions of resulting cryogel properties. The representative cryogels so prepared had high permeability and flow rates between 0.5 and 3.0 cm/min. The study demonstrated the interconnected macroporous nature and high mass transfer nature of pHEMA-PVA-BC cryogels. The work indicates that machine learning may be used to prepare cryogels of desired properties.^55^ Moreover cryogel columns owing to their excellent transport properties for the particulate matter and mechanical shape memory nature (Section 2.3) can be used for isolating living cells using elastic deformation of the cryogels. This allows release of cells upon applied stress and return of cryogel to original shape upon releasing the stress.^7,56,57^

### 2.2 Swelling Ratio and Kinetics

An interesting property of cryogels is their high osmotic stability, which corresponds to their ability to take up and hold significant quantities of water or suitable solvents. The polymeric walls and the pore architecture in cryogels allow the pore capillaries to hold a large volume of water in addition to the polymer-bound water/solvent. The water in the capillaries constitutes more than 70–75% of the total water in the cryogel, and most of this water can be easily squeezed out by slight mechanical compression without destroying the gel network.^58^ The polymer-bound water is rather firmly bound to the polymer network and can only be taken out by methods such as heating.^59^ The existence of liquid in different forms in the cryogels has been confirmed by various studies as described in Section 2.1.^60,61^ Swelling kinetics and the swelling ratio in cryogels are routinely measured to estimate the porosity and pore interconnectivity. The water uptake in the dried cryogel matrix is measured over regular intervals of time to calculate swelling kinetics and swelling ratio in the equilibrium state.^62–69^ In a recent study, the swelling capacity of the hydroxy propyl methoxy cellulose (HPMC) cryogels was shown to be dependent upon the chemical structure of the cryogels. HPMC cryogels made with a high degree of hydrophobic methyl groups showed the least degree of swelling, while the ones with hydrophilic hydroxypropyl groups showed the highest wettability and a high degree of swelling. The study shows that the water uptake capability of the cryogels can be controlled by tuning the hydrophobicity and hydrophilicity of the polymeric precursors.^70^

A study of stimuli responsive cryogels of poly­(*N*-isopropylacrylamide) (NiPAAm) or polyvinylcaprolactam by Srivastav et al.,^71–73^ showed a higher equilibrium swelling ratio and a faster equilibrium swelling and deswelling of cryogels with time (∼20 min) than the corresponding nonporous hydrogels (∼2 days) (Figure 4). The fast kinetics of swelling and higher swelling ratio again emphasize the presence of high pore volume, pore interconnectivity, and stable pore walls in the case of cryogels. Another study of poly NiPAAm-HEMA-dextran also showed similar temperature-responsive swelling and deswelling (Figure 5) of the cryogels. These cryogels were applied for controlled release of simvastatin for bone tissue engineering.^74^ The rapid response time in cryogels compared to traditional hydrogels has led to the design of stimuli responsive cryogel systems for drug delivery, wound healing, and bioseparation.^75–78^

**Figure 4:**
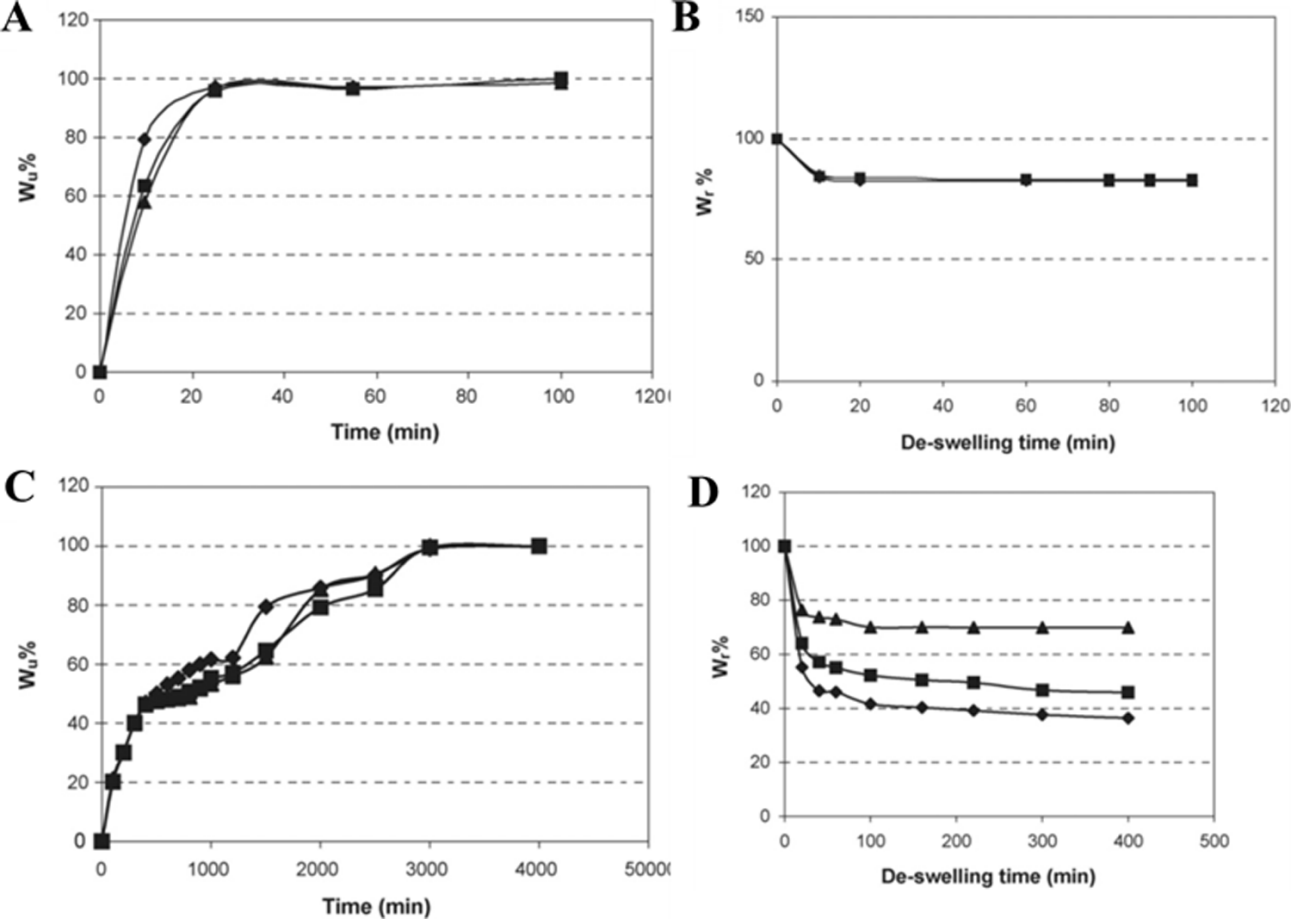
Swelling and deswelling of poly­(NiPAAm) gels over time. Water uptake capacity was measured at regular time intervals for (A) poly­(NiPAAm) cryogel and (C) poly­(NiPAAm) hydrogel. Water retention capacity was quantified at regular time intervals for (B) poly­(NiPAAm) cryogel and (D) poly­(NiPAAm) hydrogel. Gel concentrations were varied: 6, 7, and 8% and the respective cryogel and hydrogel were characterized. Note the difference in swelling rate of cryogels in A is in minutes, while hydrogels in B take hours to reach equilibrium. Adapted or reprinted in part with permission from cited ref^71^. Copyright [2007/Elsevier].

**Figure 5:**
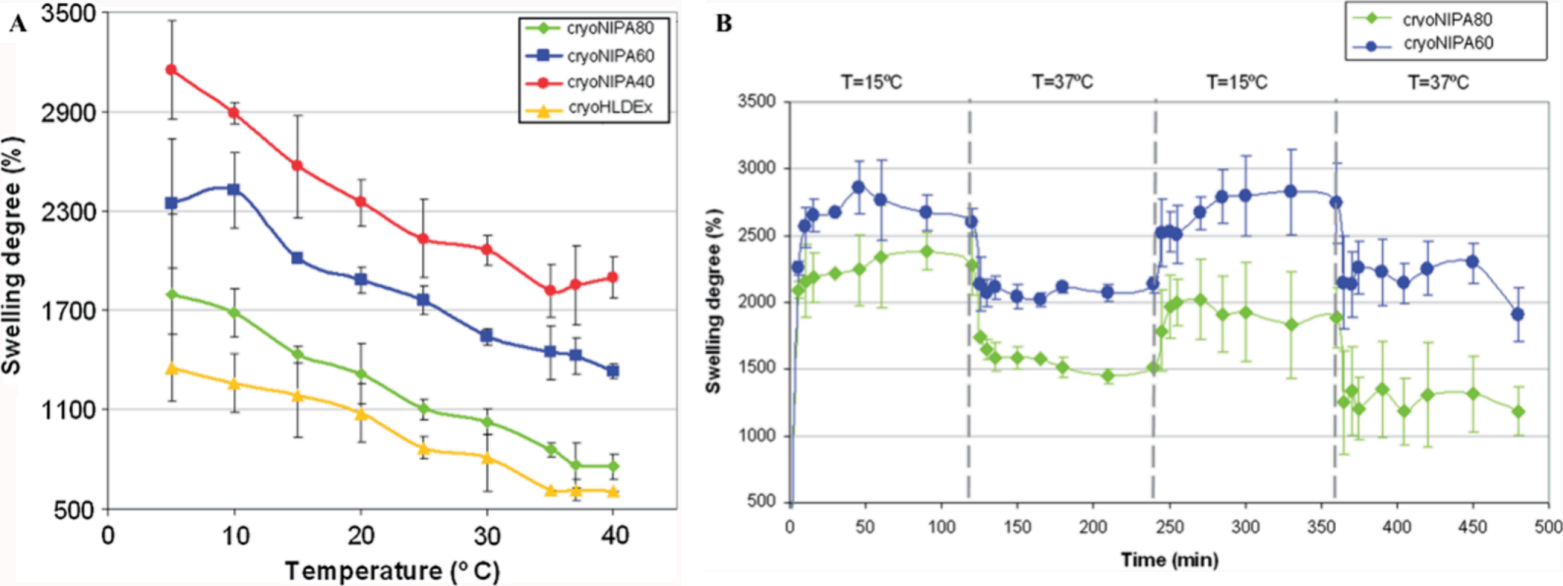
Temperature-responsive swelling characteristics of poly NiPAAm-HEMA-dextran cryogels. (A) Equilibrium swelling degree of poly NiPAAm-HEMA-dextran cryogels at varied temperatures. (B) Swelling–deswelling kinetics of poly NiPAAm-HEMA-dextran in response to temperature in water. Adapted or reprinted in part with permission from cited ref^74^. Copyright [2013/Taylor & Francis].

### 2.3 Mechanical and Shape Memory Properties of Cryogels

Mechanical properties of cryogel are important for their use in various biomedical and nonbiomedical applications. The compression and tensile strength of different cryogels have been measured using standard mechanical tests. Confined and unconfined tests of compression and tensile strength of cryogels have been done using materials testing machine by placing the sample between two parallel plates and compressing or stretching the sample, respectively. Cryogels offer superior mechanical toughness compared to hydrogels and aerogels, making them ideal for pressure-bearing applications.^79–81^ Like hydrogels, they consist of a 3D hydrophilic polymer network, but their key distinction lies in their macroporous, sponge-like structure—featuring large, open pores surrounded by thick, condensed polymer walls. This architecture enhances their toughness and resilience. Unlike fragile/brittle hydrogels, cryogels can be exceptionally soft but tough and can be significantly compressed and then return to their original shape, a property valuable for insertion and removal from tissue.^3,15,82,83^ Notably, cryogels made from the same monomer composition as hydrogels can withstand higher stress and strain before breaking, despite being three times softer.^84^ In a recent example, silicone cryogel skeletons were used to augment the survival and mechanical strength of hydrogel encapsulated cells for cell therapy, further demonstrating the resilience of cryogel structures.

Many studies have illustrated mechanically robust and elastic nature of cryogels.^67,85,86^ The modulus of cryogels ranges from several kilopascals (kPa) to several megapascals (MPa), depending on the specific material, freezing history, and other manufacturing parameters. For instance, the modulus of PVA cryogels can range from 4 to 180 kPa. Silk cryogels have been reported to have moduli ranging from 0.5 to 283 kPa. The modulus of hyaluronic acid (HA) cryogels can fall within the range of 0.2–2 kPa, while the modulus of starch-based cryogels ranges from 2.75 to 18.94 MPa.^87–91^ Moreover, many of these cryogels regain their shape even after 80% compression load, but this property is dependent upon the type of polymeric system.^67,68,92,93^

The mechanical strength or elastic modulus of cryogels varies greatly with the change in precursor concentrations, cross-linking mechanism, degree of cross-linking, and cryogenic regime.^64,66,71,91,92,94,95^ Particularly, in the instance of cryogels formed by physically cross-linked polymer networks wherein the history of the cryogenic regime of a particular sample and the thawing rate have been shown to greatly affect the elastic modulus and sponginess of the cryogels.^64,96^ Moreover, for physically cross-linked cryogels, the slower the thawing rates of the gel, the greater the mechanical resilience of the cryogel for the same initial concentration of the polymer. As exemplified by poly­(vinyl alcohol) (PVA) cryogels in which, for an initial concentration of 10% w/v PVA, and storage temperature of −20 °C the shear modulus of the gel melted at a rate of 0.03 °C min ^–1^ was 3 times (9.40 kPa) greater than the shear modulus of cryogel melted at a degree of 0.3 °C min^–1^ (3.20 kPa).^97^

In the case of covalently cross-linked cryogel systems, apart from the precursor concentration and freezing temperature, the choice of the cross-linker has shown to play a critical part in influencing the ultimate mechanical properties of the cryogels. Okay and colleagues have fabricated a range of HA cryogels using ethylene glycol diglycidyl ether (EGDE) and *N, N*-dimethylacrylamide (DMAA) as cross-linkers. The HA cryogels prepared using the DMAA cross-linker exhibit a compressive modulus of 2.6 ± 0.2 MPa, which is 80 times higher than those synthesized using the EGDE cross-linker.^91,98^ Remarkably, the HA cryogels also exhibit cartilage tissue-like poroelasticity due to flow-dependent flowing in and out of water from the pores.^91^ In contrast to HA cryogels formed through covalent cross-linking, those created solely through physical interactions possess a compressive modulus of 100 Pa.^99^ Similar effects of cross-linkers, cross-linking mechanism, and freezing temperature on the mechanical properties of the silk cryogels have also been reported by multiple groups.^66,100^

The mechanical characteristics of cryogels can be further modified by synthesizing composite cryogels composed of an interpenetrating network (IPN) for two or more cross-linked networks. Several cryogels with high mechanical strength consisting of IPN and double network have been synthesized recently.^67,101–110^ A common challenge with sequential double network cryogels is that the succeeding network occupies the pore space of the preceding network, leading to a decrease in porosity. Gong and colleagues^102^ overcame this limitation by preparing a double network cryogel by sequential cryogelation of a polyelectrolyte with high swelling capacity and a neutral polymer network. They controlled the ice nucleation for the cryogelation of the second network such that the subsequent network diffused into the gel phase of the prior and allowed the formation of interconnected pores while yielding a high compressive modulus of 100 kPa, which was 2–3 fold greater than that of the single network. Although sequential cryogelation results in high mechanical strength, the process requires multiple steps, and it is complicated. On the other hand, IPNs formed via cryogelation can be made via simultaneous cross-linking of the two polymer networks and result in increased strength of the resulting cryogels.^111^

Rheological measurement of cryogel, including storage (*G*′) and loss modulus (*G*″), can be done to measure the flow-dependent viscoelastic or poroelastic properties of cryogels, which are similar to cartilage as a load-bearing tissue. The rheological measurements have been conducted using a standard rheometer or dynamic mechanical analyzer. The viscoelastic nature of cryogels can be measured using cyclic strain-sweep or creep test experiments.^91^ Cryogels exhibit a high ratio of *G*′/*G*″ prime, indicating the formation of a completely elastic network.^112–115^ Particularly, the physically cross-linked cryogels show a polymer concentration and history of freeze–thaw cycle-dependent increase in modulus (Figure 6).^115^

**Figure 6:**
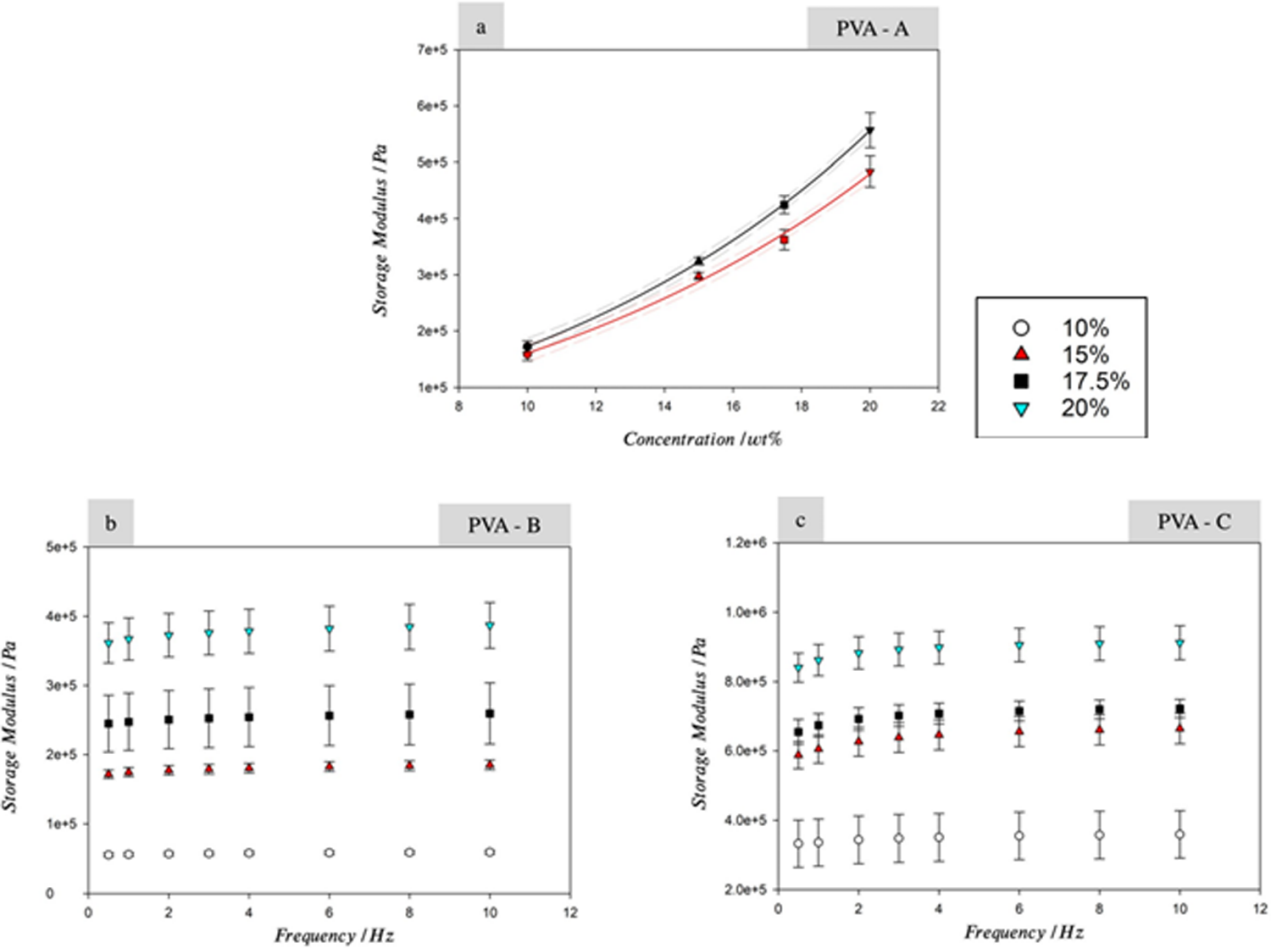
Storage modulus of cryogels vs concentration at 0.5 Hz (red) and 10 Hz (black) for PVA-A (a), PVA-B (b), and PVA-C (c) made using 1 and 3 freeze thaw cycles, respectively, at concentrations of 10, 15, 17.5, and 20% w/w. Note that PVA-C shows a higher storage modulus than PVA-B for the same concentrations. Error bars show 95% confidence intervals (*n* = 6). Adapted or reprinted in part from cited reference with permission.^115^ Copyright [2021/Elsevier].

Cryogels exhibit both viscoelasticity and poroelasticity, but these properties differ in their mechanisms and performance. Viscoelasticity originates from the movement of polymer chains, which is manifested as the time-dependent response of the material to external forces. It is typically characterized as the storage modulus (*G*′) and the loss modulus (*G*″).^116^ Poroelasticity involves the coupling behavior between the porous skeleton of the material and the liquid within it. Under the action of external forces, the liquid migrates through the pores, causing deformation and delayed recovery of the skeleton, a phenomenon typical of tissues such as cartilage.^91,117^ The polymer network structure primarily controls the former, while the latter is influenced by the pore size, porosity, and permeability. In cryogels, these two mechanical properties work synergistically to provide excellent deformation recovery, buffering energy absorption, and biofluid responsiveness, which is the key basis for their wide applications in biomedical and nonbiomedical engineering areas.

Cyclic compression testing of cryogels (a measure of fatigue resistance) has shown cryogels to be highly resistant to repeated loading under 10–60% strain without showing signs of deformation even after 100+ cycles. The fatigue resilience and excellent shape recovery properties^68^ of the cryogels are highly regulated by the pore architecture and in turn the freezing regime.^58,91–93,105,107,118^ Recently, chitosan cryogels with extremely high elasticity and shape recovery were made. The chitosan cryogel architecture was inspired by the hierarchical structure of the spider web (Figure 7A,B).^11^ The chitosan micro/nanofibers were made by shear-flow induction. Subsequent freeze-drying led to the induction of physicochemical cross-linking and the formation of a cryogel with interconnection between the micro/nanofibers. The use of hierarchical micro- and nanofibers endowed cryogels with high fatigue resistance and fast shape recovery within ∼1 s in response to water (Figure 7C–E). The study shows that use of hierarchal structures can optimize molecular interactions leading to development of cryogels with high resilience,^11^ while a lack of similar studies indicates a need to further optimize the cryogelation process to generate bioinspired hierarchal structures. These unique mechanical properties make cryogels a potential tissue engineering scaffold for bone defect regeneration,^119,120^ skeletal muscle regeneration,^105^ tissue-engineered artificial cartilage constructs,^121^ and soft robotics.^88^

**Figure 7:**
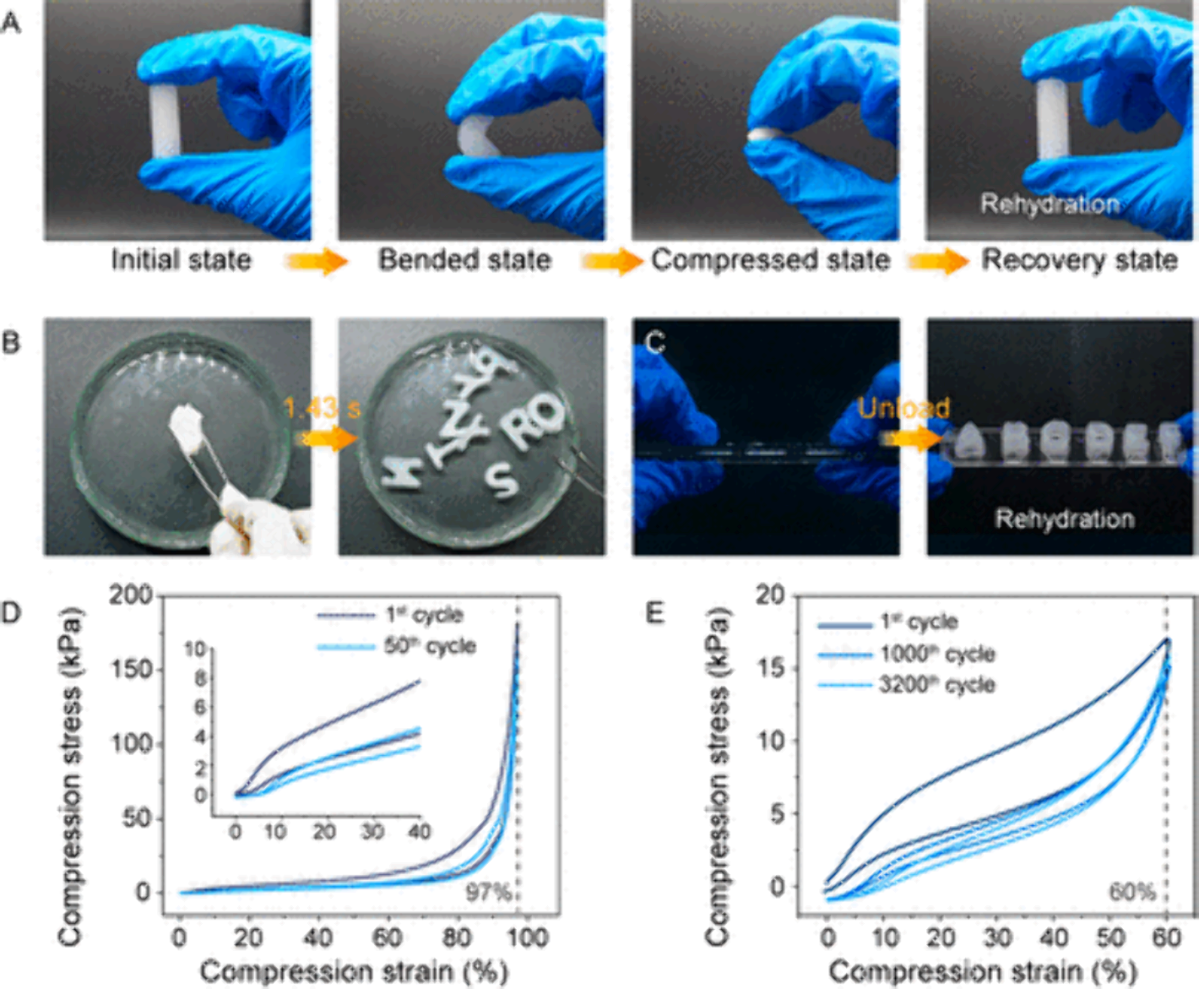
Mechanical properties of cryogels. (A–C) Images of the water-swelled chitosan hybrid micronanofiber (CMNF) cryogel for shape recovery after compression, Photograph courtesy of Luhe et al., Copyright 2023 (D, E) Stress–strain curves of the CMNF with 97% strain (50 cycles) and 60% strain (3200 cycles). Adapted or reprinted in part with permission from cited ref^11^. Copyright 2023/American Chemical Society.

### 2.4 Biological Properties

Biocompatibility, cell adhesion, and cell infiltration are some of the important biological properties of cryogel that need to be evaluated for specific biomedical applications. The biocompatibility of the cryogels is dependent upon the composition and structure of the polymeric system. In vitro biocompatibility of cryogels can be measured using standard techniques, including cell viability via LIVE/DEAD assessment using standard dyes and cell proliferation using metabolic assays at different time points after culture. Specific functional assays may be conducted to test cell function after culture on cryogels. For instance, Jain et al. showed albumin synthesis and glutathione production in liver cells cultured in a cryogel bioreactor for their application as a bridging device for patients waiting for liver transplants.^110^ Zhang et al. showed production of cartilage-specific ECM synthesis and gene expression in mouse mesenchymal cells cultured in cryogels for 14 days.^121^ Similarly, Wei et al. showed cells showing bone-specific gene expression and differentiation of the recruited cells in the defect area.^122^ While the biocompatibility of polyaniline cryogels made up with conducting materials was tested using embryonic stem cells capable of differentiating into beating cardiomyocytes when cultured within the cryogels.^123^ A common challenge in applying image-based assays to cryogels is their opaque, dense structure, which hinders the deep visualization of cells throughout the scaffold. Typically, this requires processing and sectioning of the samples. Advanced optical imaging modalities such as spinning disk confocal microscopy or computationally enhanced (deconvolution) wide-field systems can partially overcome these limitations. These techniques facilitate improved optical sectioning and deeper imaging penetration without the need for extensive sample preprocessing. Similarly, colorimetric and molecular assays require complete enzymatic, mechanical, or chemical degradation of the cryogel matrix to release embedded cells, nucleic acids, or proteins. This often necessitates additional steps that may result in sample loss or incomplete recovery due to residual entrapment within the gel network. As a result, conventional assays must often be adapted or customized for reliable analysis in 3D cryogel systems.^121^

Cryogel biocompatibility varies widely depending on the polymer system. Natural polymer-based cryogels (e.g., gelatin, chitosan, and hyaluronic acid) closely mimic the extracellular matrix (ECM), promoting strong cell adhesion and tissue integration, but they typically lack mechanical strength. In contrast, synthetic cryogels (e.g., PVA, PEG, PAAm) offer excellent mechanical robustness and tunable properties, yet lack inherent bioactivity. To enhance biocompatibility, synthetic cryogels can be physically coated with ECM components like collagen, decellularized matrix, or Matrigel.^124^Alternatively, they can be chemically functionalized with bioactive substances—such as ECM-mimetic peptides (e.g., RGD, GHK, YIGSR) or growth factors (e.g., VEGF, FGF, BDNF)^125,126^ -either before or after gelation. However, most studies to date focus primarily on RGD peptides, with limited exploration of other ligands.^127–130^ Composite cryogels combining natural and synthetic polymers (e.g., polysaccharides, heparin) provide a promising strategy to integrate bioactivity with mechanical strength, expanding their potential in tissue engineering and regenerative medicine.^101,111,121^

In addition to the polymer used for cryogel synthesis, the highly hydrated structure of cryogels confers additional biocompatibility for tissue engineering applications.^3,7^ The presence of >90% water on the surface of scaffolds has been shown to make them highly compatible and less susceptible to protein fouling.^131^ Studies evaluating in vitro and in vivo biocompatibility of various cryogel formulations confirm their compatibility with cultured cells as well as surrounding tissue upon implantation.^124,132,133^ In vitro studies show sustained cellular growth upon culture on cryogel scaffolds when compared to 2D counterparts in flat bottom culture plates.^92,109,111,134,135^ These studies indicate the availability of a very high surface area in cryogels for maintenance of a high-density cell culture. The interconnected macropore-based capillary network in cryogels resembles the fiber capillary of the hollow fiber bioreactor. Further, the pore walls support high cell density culture due to greater surface area.^136^ Cryogels present a high surface area and is assessed to be 4.3 m^2^ g^–1^, which is ∼70 times higher compared to the surface area of 0.6 m^2^ g^–1^ provided by PAAm microbeads (0.2 mm). Cells are usually seeded directly over cryogels, without any pre-equilibration with culture medium, which is an added benefit over conventional hydrogels that need to be equilibrated with medium before cell seeding. Additionally, cells can be seeded at a high flow rate into the cryogel scaffold owing to the high porosity and flow rates of the cryogels. Moreover, these properties of the cryogel also allow for efficient cellular infiltration. Upon seeding, the cells can penetrate through the scaffold and get entrapped in the interior between the pores.^92,109,111,124,134,135^ Excellent cell infiltration in macroporous cryogels has led to their use as model platforms for studying tumor-associated macrophage invasion and their targeting.^63,137^

In vivo studies of cryogel implantation show stable integration of the implanted cryogel with the adjacent tissue, while the porous structure allows for cellular infiltration into the implant.^69,110,135^ In vivo evaluation of cryogels typically involves implanting sterile samples of defined dimensions at injury sites in animal models. Biocompatibility is assessed through macroscopic observation, histological analysis, immunohistochemistry for inflammatory markers, and serum analysis for proinflammatory cytokines. Most studies have focused on short-term biocompatibility, ranging from one to 4 weeks. Moreover, there are only a few longitudinal studies evaluating the in vivo performance and biodegradation of cryogels in live animals under physiological conditions.

In one of the studies, cryogels composed of conductive polymers—polypyrrole and carbon nanotubes—were used as penetrating electrodes for brain stimulation. These cryogels demonstrated excellent in vivo stability and biocompatibility over 4 weeks, with no detectable inflammation. Electrical stimulation via the cryogel electrodes significantly increased neural precursor cell (NPC) populations in ex vivo brain tissue as well as in vivo models.^138^ Another pioneering study used alginate cryogels embedded with bioluminescent reporter cells, showing improved cell survival, retention, and prolonged engraftment at the injection site compared to conventional injection methods.^83^

To longitudinally assess in vivo biodegradability, hyaluronic acid (HA) cryogels synthesized with oxidized HA and glycidyl methacrylate were injected subcutaneously into mice. These cryogels degraded within 2 weeks, as confirmed by ultrasound imaging in live mice, and enhanced antigen (ovalbumin) release and uptake by immune cells.^139^

Longer-term in vivo studies of cryogels are limited but promising. Gelatin-HA cryogels implanted in mice and pigs for adipose tissue engineering showed high levels of leptin-positive cells after 8 weeks.^140^ Gelatin-chitosan and gelatin-heparin double cryogels were used to sequentially release growth factors, promoting cranial defect healing in mice at 8 and 12 weeks. Moreover, these studies showed minimal to no inflammatory signs at the site of injury.^125^ Together these studies indicate the important role of high porosity, surface area, and open porous structure for using cryogel for tissue engineering applications.

### 2.5 Injectability of Cryogels

It is preferable to use injectable hydrogels for biomedical applications rather than invasive operations to lower the chance of infection.^133^ Cryogels’ elastic structure and rapid shape recovery properties allow for minimally invasive injection, leading to successful implantation in tissue regeneration. Injectable cryogels are becoming increasingly popular in the biomedical field over the past decade.^85,141,142^ The distinctive interconnected macroporous structure makes cryogel appropriate for residing and protecting host cells during injections and provides a favorable milieu for cell infiltration and the creation of new blood vessels. An easy and straightforward approach to test cryogel injectability is to load cryogels in a conventional syringe and then evaluate changes in cryogel properties and shape and integrity before and after injection. Cryogel injectability can also be tested using flow test mode with varying shear rates.^143^

Injecting cryogels for biomedical treatment has recently been found to alleviate pain and reduce the risk of infection in patients undergoing surgical procedures. For instance, cryogel-based vaccines were developed to activate the immune cells in melanoma.^144^ The cryogels were subcutaneously injected, allowing cell migration into the scaffold as well as activation of dendritic cells, thus stimulating the immune system against melanoma.^144^

Large cryogels (up to 8 × 8 × 1 mm) can be delivered with 16G needles, providing a less invasive option than surgery. Further progress has been made in enhancing injectability by reducing polymer concentration—for example, lowering the polymer concentration in gelatin cryogels enabled their passage through narrower 17G needles without compromising mechanical integrity or biological function.^145,146^ These advances underscore the potential of injectable cryogels in applications such as regenerative medicine and wound healing.

However, challenges remain in minimizing tissue damage and pain during delivery. Although 16G or even 17G needles are considered less invasive than surgical methods, they can still cause significant tissue trauma, especially in delicate or sensitive regions, such as the brain or peripheral nerves. For such applications, smaller gauge needles (≥25G) are preferred. Bulk cryogels, due to their size and stiffness, are currently not compatible with these finer needles. To address this limitation, microengineered cryogels, or microcryogels, have been developed. For instance, ∼300 μm microcryogels were created, which retained structural integrity and supported high neuron-like cell viability after injection through a 27G needle.^147^

Despite these promising results, several critical challenges remain, including enhancing the compressibility of cryogels to allow large-size cryogels to be passed through fine-gauge (<21G) needles without losing functional or structural properties. Additionally, fabricating cryogels with precise, preset geometries to conform to irregular tissue defects and ensure complete space-filling and functional integration upon injection will be key to establishing the complete translational potential of cryogels.

### 2.6 Anisotropic Properties/Unidirectional or Directional Freezing

Cryogels with anisotropic properties can be obtained with directional or unidirectional freezing. This method has been used to generate cryogels of anisotropic mechanical strength, swelling ratio, pore size, and shape influenced by the direction of freezing, thus displaying tissue-like anisotropic behaviors. The freezing rate is the major determining factor in the fabrication of aligned macropores. As such, the faster freezing rate in the desired direction should be faster than in the other direction to resulting in aligned macropores in the direction of freezing.^7,105^ Cryogels with unidirectional freezing have been made using a variety of polymers, including silk,^58^ silk-cellulose,^148^ chitosan-gelatin,^149^ polyethylene glycol (PEG),^150^ etc. A recent study by Okay and colleagues generated anisotropic silk fibroin cryogels. The group utilized two different methods to generate silk cryogels with anisotropic properties. In the first method, the silk fibroin cryogels were prepared by combining a directional precooling step with cryogelation and exhibited a high degree of anisotropic properties.^151^ They further reported an improved second method to obtain cryogels with anisotropic properties using a customized reactor with a copper bottom and Teflon mold, leading to high thermal conductivity. The reactor allowed the generation of aligned pores by freezing of the copper bottom in liquid nitrogen and subsequent cryogelation at −18 °C. The silk fibrin cryogels so obtained showed the highest modulus anisotropy of 21 with moduli of 2.3 MPa in parallel and 0.11 MPa in perpendicular to the freezing direction.^58^ Kumar et al. have developed chitosan-gelatin cryogel fillers via directional freezing for tissue engineering of peripheral nerve.^149^

Guo et al.,^152^ prepared PAAm and PNiPAM cryogels coupled to DNA aptamers via unidirectional freezing. This led to anisotropic properties of mechanical strength, swelling ratio, and efficient capture of biomolecules. The authors demonstrated that unidirectional pores increase the responsiveness of cryogels to biomacromolecules. Further, the unidirectional macropores facilitated cell migration, leading to efficient cell capture by the functionalized aptamer and cell release due to the thermosensitive nature of the cryogel.

The cryogels with aligned pores have been shown to facilitate cell migration and accelerate tissue regeneration. Aligned cryogel microfibers were combined with a 3D-printed gelatin scaffold. The cryogel fibers were further functionalized with a vascular endothelial growth factor (VEGF) and bone morphogenetic protein (BMP) mimicking peptide. Compared to random cryogel fibers, the aligned cryogel microfibers along with the peptides promoted cell infiltration and faster bone regeneration of cranial defect in a mouse model.^122^

### 2.7 Future Directions and Emerging Applications: Expanding the Cryogel Functionality beyond Traditional Barriers

Cryogels have emerged as promising platforms in biomedical engineering, but their full potential remains underexplored. The inherent properties of cryogels—such as macroporosity, mechanical tunability, injectability, and responsiveness to stimuli—make them suitable for a variety of advanced and unconventional applications. However, several challenges remain, such as designing cryogels of complex shape or generating cryogels with high compressibility and macroporosity. The combination of cryogelation with the advancements in biomaterial fabrication technologies can further enhance cryogels physical properties and significantly expand their utility in biomedical and nonbiomedical fields.

#### 2.7.1 Fabrication of Cryogels of Complex Shape and Organized Architecture Combination of Cryogel and 3D Printing

Conventional cryogel fabrication often yields random, nonuniform structures, which cannot be readily tailored for applications requiring precise design.^10,153^ However, integrating cryogelation with additive manufacturing, particularly 3D printing, presents a powerful strategy to overcome these limitations. This combination permits for the fabrication of scaffolds with precisely defined architectures, better mimicking the ECM and enhancing cellular adhesion, viability, and differentiation.^154,155^ Additionally, 3D printing can create patient-specific cryogels necessary for personalized medicine and implantable devices.^156–158^

3D printing technology offers enhanced precision and capability to design complex shapes with intricate geometries, constructing structures layer by layer.^159^ Various 3D printing techniques enable the fabrication of 3D cryogenic scaffolds featuring interconnected macropores, complex geometries, and spatial control over macroporosity.^86^ These methods include extrusion processes on cold platforms^158,160,161^ and within cryogenic chambers, light-assisted cryoprinting, cryogelation after printing,^162^ and the incorporation of cryogel precursors into another 3D-printed scaffold.^122,163,164^ For example, Bilici et al.^165^ utilized gelatin methacrylate-alginate low viscosity inks in a nanoclay support bath to produce stable, photo-cross-linked cryogel structures, while Cheng et al. demonstrated the modular assembly of cryogel structures 5 times taller than starting modules using self-healing dual-network bioinks.^67^ A summary of cryoprinting approaches and their corresponding materials and applications is shown in Table 1, and a schematic of cryoprinting methods is illustrated in Figure 8. These advancements suggest that cryoprinting will be instrumental in developing next-generation scaffolds for personalized medicine, offering high structural fidelity, modularity, and tunable mechanics.

**Figure 8:**
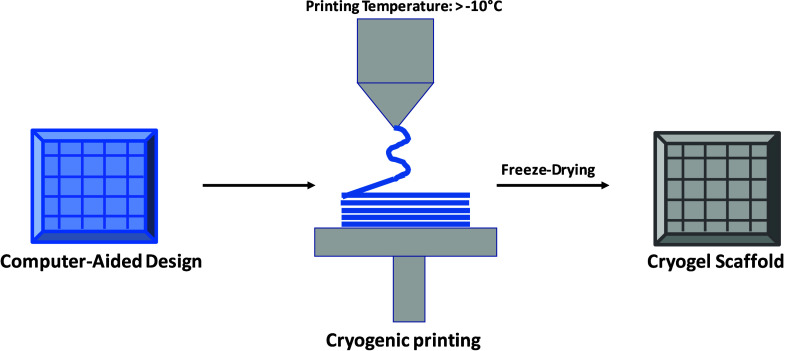
Schematic representation of the cryoprinting method (redrawn inspired by cited reference).^166^

**Table 1:** Tabulated Representation of Certain Examples of Polymer Types, Printing Methods, and the Associated Applications of Cryoprinting

**type of polymers**	**printing method**	**applications**	**references**
gelatin methacrylate, alginate	embedded printing within a shear thinning bath	tissue engineering	^165^
β-tricalcium phosphate (β-TCP)/poly(lactic-*co*-glycolic acid)-polycaprolactone	microextrusion-based cryogenic 3D printing	bone tissue engineering and antibacterial activity	^166^
laponite, silk-fibroin	light-based (405 nm, 40 s) cryoprinting	bone tissue engineering	^167^
agar and alginate	temperature-controlled cryoprinting	tissue engineering	^168^
poly(3,4-ethylenedioxythiophene):poly(styrenesulfonate)-poly(vinyl alcohol) (PEDOT:PSS–PVA)	multimaterial cryogenic printing	biomimetic heart valves	^169^
alginate, calcium carbonate and d-glucono-δ-lactone (GDL)	3D extrusion-based cryoprinting	drug delivery	^170^

#### 2.7.2 Nanoparticle and/or Drug Incorporated Cryogels

Another exciting avenue is the incorporation of nanoparticles and/or therapeutic agents within cryogels to create multifunctional systems that can enhance their existing applications or expand applications in new directions. The highly porous structure and water content of cryogels allow for the effective loading and controlled release of a variety of cargoes, ranging from small molecule drugs to large nanoparticles. For instance, magnetic nanoparticle-loaded cryogels have been explored for hyperthermia-mediated drug release,^171^ while silver and iron oxide nanoparticles have shown promise in antibacterial, catalytic dye removal,^172^ and environmental (heavy metal ion isolation)^173^ applications, respectively. Cryogels can also be engineered for targeted release by conjugating drugs directly to the matrix or embedding bioactive agents within nano/microscale architectures.^174^ Further, embedded nanoparticles can augment cryogel injectability,^175^ 3D printability,^176^ or cellular responses.^167^

While the functional enhancement is significant, challenges such as nanoparticle-induced cytotoxicity,^177^ changes in synthesis parameters, and interference with physical properties and network strength require careful consideration in future designs.

#### 2.7.3 Cryogels for Soft Robotics and Biosensors

Cryogels have broad application prospects in various emerging fields, including bioelectronics and soft robotics, due to their exceptional properties such as their capability to conform to dynamic surfaces, recover quickly from deformation, and swift stimuli-responsiveness, making them ideal candidates for wearable sensors, skin-like electronics, and implantable devices.^178^ In the field of bioelectronics, they are expected to be used in the manufacture of stretchable, self-healing, and durable electronic devices. Recent studies have demonstrated cryogel-based electronic materials capable of self-healing, stretchability, and environmental monitoring. For example, flexible and conductive cryogels with self-healing, adhesivity, and stretchability were developed by printing for real-time monitoring in plant systems, achieving long-term and stable data collection^179^ and highlighting their adaptability and functional robustness. Advances in controlling cryogel micro- and macroarchitecture—such as freezing-point manipulation and polymer aggregation—have enabled the tuning of ionic conductivity and mechanical response and achieving skin-like softness which are critical for such applications. Cryogenic multimaterial printing methods with freezing-induced solvent phase transition, instant ink gelation, and in-synch cross-linking have been developed to construct geometrically complex multimaterial 3D hydrogel machines. The 3D-printed multimaterial hydrogel soft machines have high aspect ratios and can perform versatile functions such as turbine robots capable of transportation and stimuli-responsive heart valves.^169^

## 3 Conclusions

Cryogels represent a highly promising class of biomaterials, distinguished by their facile and mild fabrication processes, inherent macroporosity, mechanical robustness, and exceptional biocompatibility. These features make them potential candidates for a broad range of biomedical and environmental applications, including tissue engineering, drug delivery, biosensing, and soft robotics.

Despite notable advancements, several key limitations must be resolved to fully harness the potential of cryogels. One major limitation is the absence of standardized, systematic approaches to monitor the dynamic structural and functional evolution of cryogels under physiological conditions. A comprehensive knowledge of the structure–function association is currently insufficient, limiting the rational design of application-specific cryogel systems. Additionally, although 3D printing has shown promise in cryogel fabrication, the seamless integration of cryogelation into scalable and reproducible bioprinting workflows remains underdeveloped. Establishing standardized protocols will be critical for fabricating patient-specific constructs with complex, nonstandard geometries.

Another pressing challenge lies in optimizing the mechanical performance of cryogels for various biological environments. This includes developing hierarchical structures that enhance mechanical strength without compromising porosity, improving mold thermal conductivity for uniform gelation and controlled directional freezing, and fine-tuning the interplay between microstructure and cell behavior to elicit tissue-specific responses. Enhancing cryogel injectability also remains a priority, requiring improved understanding and control over their mechanical and shape-memory properties to boost compressibility and recovery.

Looking ahead, future innovations should focus on the engineering of programmable cryogel systems with tunable physical and chemical features. Integrating high-throughput characterization techniques with machine learning and computational modeling will offer predictive control over cryogel behavior and accelerate their translation from laboratory prototypes to clinical and industrial solutions. In parallel, new synthesis strategies are needed to produce cryogels capable of rapid, force-responsive volume changes, overcoming the inverse relationship between porosity and swelling capacity. Expanding the material palette to include a wider array of polymeric biomaterials will further support the customization of cryogels for diverse biological contexts.

In summary, cryogels are poised to evolve beyond their traditional role as scaffolds into multifunctional platforms capable of addressing complex biomedical and environmental challenges. Bridging current knowledge gaps and leveraging emerging technologies will be pivotal in unlocking their full potential across a wide array of scientific and engineering applications.

## Data Availability

No new data were created or analyzed in this study.
